# Treatment of gestational choriocarcinoma and massive ascites by hypothermic intraperitoneal perfusion chemotherapy guided by ultrasound followed by cytoreductive surgery

**DOI:** 10.12669/pjms.292.2981

**Published:** 2013-04

**Authors:** Ming-Chen Ba, Hui Long, Yin-Bing Wu

**Affiliations:** 1 Ming-Chen Ba, PhD, Intracelom Hyperthermic Perfusion Therapy Center, Cancer Hospital of Guangzhou Medical College, Guangzhou 510095, P.R. China.; 2 Hui Long, PhD, Department of Pharmacy, Guangzhou Dermatology Institute, Guangzhou 510095, P.R. China.; 3 Yin-Bing Wu, PhD, Intracelom Hyperthermic Perfusion Therapy Center, Cancer Hospital of Guangzhou Medical College, Guangzhou 510095, P.R. China.

**Keywords:** Gestational choriocarcinoma, Hypothermic, Intraperitoneal perfusion, Chemotherapy, Malignant ascites, Ultrasound

## Abstract

A 33-year-old woman with very poor health status was admitted to our hospital because she had experienced increasing abdominal distention for three months, CT examination showed a right ovarian tumor together with massive abdominal and pelvic fluid. The patient was first treated by continuous circulatory hypothermic intraperitoneal perfusion chemotherapy (HIPC) guided by B-mode ultrasound, followed by cytoreductive surgery (CRS) after her ascites was controlled and her health condition improved. She was diagnosed with gestational choriocarcinoma (GC) based on the pathological examination of the hysterectomy specimen. She is still alive with very good health today. We think it may be a good choice for a patient in very poor health with GC accompanied by massive ascites to perform HIPC guided by B-mode ultrasound firstly, followed by CRS when the ascites has relieved and the patient’s health has improved.

## INTRODUCTION

Gestational choriocarcinoma (GC) usually arises in the uterine cavity and is associated with coincident or antecedent pregnancy.^1^ GC accompanied by massive ascites that affects patient vital signs present challenging issues in the clinical setting. The objective of the current study was to develop a successful treatment strategy for a patient in very poor health with GC accompanied by massive ascites.

## CASE PRESENTATION

**Fig.1:**
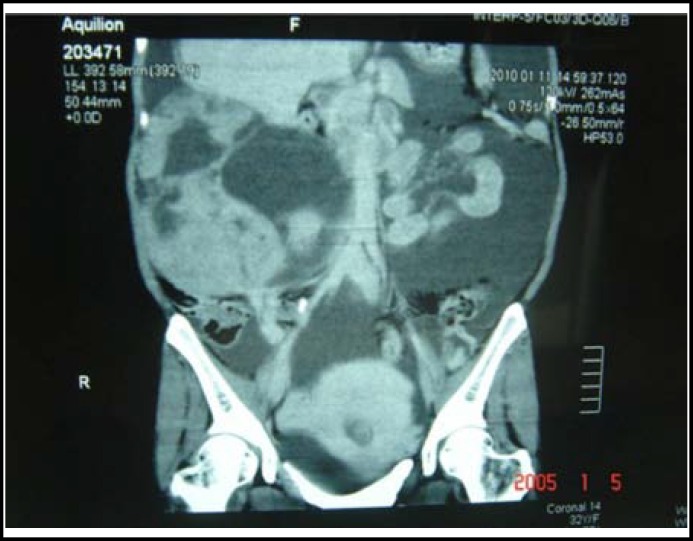
Computed tomography examination showed a right ovarian tumor with a size of 17.3 cm × 16.2 cm ×12.3 cm accompanied by significant abdominal and pelvic fluid

A 33-year-old woman who was five months pregnant, gravida six, para three, was admitted to our hospital because she had experienced increasing abdominal distention for three months. Her last menstrual period was on August 28, 2009. After two months of amenorrhea, the patient presented with increasing abdominal distention, and the following levels of serum tumor markers abnormality: beta-human chorionic gonadotropin (hCG) 7.485 mIU/ml, ovarian cancer associated antigen (CA125) 857.70 u/ml and alpha-fetal protein (AFP) 20,558.33 ng/ml. Computed tomography (CT) examination showed a right ovarian tumor with a size of 17.3 cm 16.2 cm 12.3 cm accompanied by a large volume of abdominal and pelvic fluid, and a pregnancy that appeared to have ended at four months and stillbirth (Fig.1). The patient, who was five months pregnant, was diagnosed with a right ovarian tumor, massive ascites, and stillbirth. 

**Fig.2:**
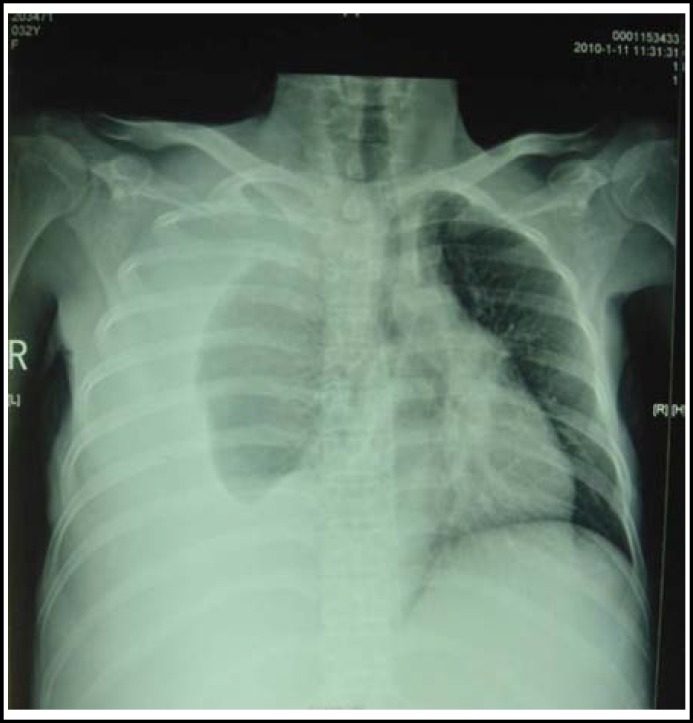
Chest X-rays examination showed a lot of right pleural effusion

After amniotic sac puncture induction of labor by B-mode ultrasonic monitoring was performed to recover the deceased fetus, the patient’s abdominal distention became even more serious after labor was induced, she presented with difficulty breathing also, and chest X-rays examination showed a lot of right pleural effusion (Fig.2), with a heart rate 130–140 beats/min, SpO2 of 92–94% in oxygen inhalation state and serological albumin (ALB) tests showing 14 g/l. After consultation with the doctors involved with her case, we developed a treatment strategy. This is, continuous circulatory hypothermic intraperitoneal perfusion chemotherapy (HIPC) guided by B-mode ultrasound was performed firstly, cytoreductive surgery (CRS) was performed after her ascites was under control and her health condition improved post HIPC follow up. Informed consent to perform CHIPC approach was obtained from the patient and followed all legal principals. This treatment strategy was approved by Medical Ethics Committee of Cancer Hospital of Guangzhou Medical College.

HIPC was performed on January 21, 2010. The puncture points were selected at regions with enough ascites in the left upper quadrant, right upper quadrant, left lower quadrant and right lower quadrant, as determined by B-mode ultrasound examinations.

Total three HIPC sessions were performed every other day, for continuous circulatory perfusion 90 minutes with a liquid perfusion velocity of 450–600 ml/min, and an inflow temperature of 43 C. Based on patient amount of ascites (3.8 l) and weight (46 kg), 0.9% saline solution (4.5 l) was used as the perfusion liquid, and the chemotherapeutic drugs cisplatin (DDP, 30 mg) and etoposide (VP16, 100 mg) were added into perfusion liquid for each of the three HIPC sessions.

After HIPC, the patient’s health status improved, and her ascites disappeared completely, pleural effusion also disappeared by paracentesis to release fluid. On February 6, 2010, we performed CRS on the patient. In her right ovary, we found an 18 cm × 16 cm ×12 cm tumor, no other tumors or peritoneal carcinomatosis sites were found in her abdominal cavity, so we performed a hysterectomy, bilateral salpingectomy, bilateral adnexectomy and greater omentectomy. Histological sectioning of the uterine endometrium showed that the tumor comprised of cytotrophoblast and syncytial trophoblast tumor cells, clear cell atypia, marked nuclear and cellular atypia and a lack of interstitial and vascular cells. Based on these findings, the patient was diagnosed with GC, right metastatic ovarian cancer and massive ascites, right pleural massive effusion and hypoproteinemia.

The patient had a good postoperative course and was discharged on the fifth postoperative day. She received systemic chemotherapy with DDP, VP16 and Bleomycin (BLM) for two circles since latter. As of September 2011, the patient is still alive and remains in very good health.

## DISCUSSION

The overall incidence of choriocarcinoma in recent years has decreased dramatically as socioeconomic conditions have improved.^1^ GC accompanied by massive ascites accumulation is rare, and treatment of gestational choriocarcinoma and massive ascites by CRS post HIPC guided by ultrasound have not been reported in the literature to our knowledge.

In recent years, HIPC present better efficacy to reduce malignant ascites and to ameliorate related symptoms. Some authors have suggested that HIPC may improve the quality of life (QOF) for patients with massive ascites who cannot undergo CRS or who are deemed unsuitable for CRS temporarily.^2^ HIPC can effectively mitigate malignant ascites, while CRS can remove bulky tumors. Thus, combination HIPC with CRS should be useful for treating patients with GC accompanied by massive ascites. Laparoscopic HIPC, which has the advantage of minimally invasive surgery (MIS), has recently been proposed as a palliative tool for patients affected by malignant ascites from peritoneal carcinomatosis of both digestive and extra-digestive origins excluded from CRS.^3^ Ultrasound examination has the advantage of high specificity in the diagnosis of liquid in the peritoneal cavity.^4^ Clinical doctors routinely place peritoneal drainage tubes guided by B-mode ultrasound to release ascites and mitigate the associated increase in abdominal distention.^4^ Based on our successful experience with laparoscopic-assisted HIPC, we employed HIPC guided by B-mode ultrasound to treat patients with ascites induced by malignant tumors, and achieved satisfactory therapeutic effects.^5^

Massive ascites not only affects QOF, but also negatively impacts respiratory, circulatory and renal function, which could lead to increased heart rate, decreased oxyhemoglobin saturation, decreased blood pressure and renal function insufficiency.^3^^,^^4^ Under these circumstances, patients cannot tolerate endotracheal anesthesia and CRS, and the risk associated with surgical treatment is very high. Treatment of malignant ascites with HIPC guided by B-mode ultrasound can achieve satisfactory therapeutic effects. After ascites are under control, the health condition and vital signs of patients will improve, which will in turn decrease the risks associated with anesthesia and CRS. In the current study, we performed HIPC guided by B-mode ultrasound first, CRS was followed after the patient’s ascites were under control and her vital signs were stable. HIPC guided by B-mode ultrasound extends the treatment window for patients with malignant ascites and permits recovery from massive ascites prior to CRS, which has good therapeutic effects.

Careful selection of the indication of HIPC guided by ultrasound in the treatment of malignant ascites should be emphasized. In order to avoid any delay in the timing of surgical treatment, patients with peritoneal carcinomatosis of unknown primary lesion status, and those who have not been treated surgically should undergo exploratory laparotomy as soon as possible after HIPC guided by B-mode ultrasound and adjuvant chemotherapy.

## CONCLUSION

HIPC guided by B-mode ultrasound can take the advantage of the opportunity for MIS. For a patient in very poor health with GC accompanied by massive ascites, it may be advantageous to perform HIPC guided by B-mode ultrasound firstly, followed by CRS when the ascites has relieved and the patient’s health has improved.
